# Effects of steroids and angiotensin converting enzyme inhibition on circumferential strain in boys with duchenne muscular dystrophy: a cross-sectional and longitudinal study utilizing cardiac magnetic resonance imaging

**DOI:** 10.1186/1532-429X-13-S1-P284

**Published:** 2011-02-02

**Authors:** Katelyn Williams, Kan N Hor, Wojciech Mazur, Hussein R Al-Khalidi, Eugene S Chung, Linda S Cripe, Kathi Kinnett, Michael D Taylor, Nandakishore Akula, William M Gottliebson, D Woodrow Benson

**Affiliations:** 1 CCHMC, Cincinnati, OH, USA; 2 The Heart and Vascular Center at The Christ Hospitals, Cincinnati, OH, USA; 3 Duke University School of Medicine, Durham, NC, USA

## Introduction

**S**teroid use has prolonged ambulation in Duchenne muscular dystrophy (DMD) and combined with advances in respiratory care overall management has improved such that cardiac manifestations have become the major cause of death. Unfortunately, there is no consensus for DMD-associated cardiac disease management.

## Purpose

To assess effects of steroid use alone or in combination with angiotensin converting enzyme inhibitors (ACEI) or angiotension receptor blocker (ARB) on cardiac magnetic resonance imaging (CMR) derived circumferential strain (ε_cc_).

## Methods

We reviewed our DMD CMR database and medical records from February 2006 to 2010. The study cohort was divided into patients receiving steroids alone (Group A) or steroids plus ACEI or ARB (Group B). Analysis of covariance was used to assess the effect of medication on heart rate (HR), left ventricular ejection fraction (LVEF), mass (LVM), end diastolic volume (LVEDV) and ε_cc_ with age as a continuous covariate.

## Results

A total of 206 studies from 136 DMD subjects were included in the analysis. Group A (114 studies) was younger than Group B (92 studies)(10±2.4 vs. 12.4±3.2 years, p< 0.0001). However, HR, LVEF, LVEDV and LVM were not different between the two groups (Table 1). ε_cc_ magnitude was significantly lower in Group B (-13.8±1.9 vs -12.8±2.0, p= 0.0004), but age correction using covariance analysis eliminated this effect (Table 2). After mean follow-up of 15 months, ε_cc_ of neither group improved compared with baseline (Table 3).

**Table 1:** DMD Patients characteristics

Parameter	Steroid Only (A) (n=114)	Steroid plus ACEI_ARB (B) (n=92)	P-value
Age (yrs)	10.0 ± 2.4	12.4 ± 3.2	<0.0001
Heart Rate (bpm)	101 ± 19	104 ± 15	0.2498
LVEDV (mL)	82.5 ± 21.8	86.7 ± 24.8	0.2023
LVM (g)	58.6 ± 19.4	62.1 ± 19.8	0.1031
EF (%)	64.2 ± 6.1	62.8 ± 7.5	0.1414
ε_cc_ (%)	-13.8 ± 1.9	-12.8 ± 2.0	0.0004
Steroid dose (gram/kg/day)	0.7 ± 0.29	0.6 ± 0.22	0.4838
ACE-I dose (gram/kg/day)	N/A	0.16 ± 0.08	N/A
ARB dose (gram/kg/day)	N/A	0.73 ± 0.29	N/A

Abbreviations: ACE-I = Angiotension Converting Enzyme Inhibitor, ARB = Angiotension Receptor Block, bpm = beat per minute, Clinic Prior to CMR = Previous Clinic Visiting Documenting Medication and Dose Prior to Cardiac Magnetic Resonance Imaging Study, DMD = Duchenne Muscular Dystrophy, ε_cc_ = Circumferential Strain, EF = Ejection Fraction, LVEDV = Left Ventricular Endiastolic Volume, LVM = Left Ventricular Mass.

**Table 2:** Analysis of covariance summary results: Comparisons between steroid only vs. steroid plus ACEI_ARB (medication) adjusted for age as continuous variable.

	Medication		Age	
Response Variable	F-statistics	P-value	F-statistics	P-value

Heart Rate (bpm)	1.11	0.2930	0.00	0.9979
LVEDV (mL)	1.33	0.2503	37.01	<0.0001
LVM (g)	2.19	0.1405	64.38	<0.0001
EF (%)	0.03	0.8594	9.10	0.0029
ε_cc_ (%)	1.74	0.1885	30.85	<0.0001

Abbreviations: ACE-I = Angiotension Converting Enzyme Inhibitor, ARB = Angiotension Receptor Block, bpm = beat per minute, ε_cc_ = Circumferential Strain, EF = Ejection Fraction, LVEDV = Left Ventricular Endiastolic Volume, LVM = Left Ventricular Mass.

**Table 3:** DMD Serial Study Characteristics

Group	Group A (Steroids)			Group B (Steroids plus)			Group A to B		
Number of patients	n=28			n=31			n=11		
Time Interval (mo)	Mean=14.9 ± 5.6; range = 8.5-29.7			Mean = 15.1 ± 5.9; range 8.4-35.8			Mean = 15.6 ± 5.9; range = 5.8-25.5		
CMR Study	Study 1	Study 2	P-value	Study 1	Study 2	P-value	Study 1	Study 2	P-value
Age (yrs)	9.30 ± 1.5	10.5 ± 1.6	<0.005	11.7 ± 3.4	12.97 ± 3.4	0.148	10.8 ± 2.5	12.0 ± 2.2	0.252
HR (bpm)	101 ± 21	99 ± 16	0.762	105 ± 14	105 ± 15	0.996	100 ± 14	102 ± 18	0.682
LVEDV (mL)	82.7 ± 19.3	86.5 ± 21.6	0.494	84.9 ± 29.2	90.0 ± 30.8	0.499	85.6 ± 18.6	81.2 ± 15.6	0.556
LVM (g)	57.1 ± 15.1	57.7 ± 16.9	0.890	60.9 ± 21.4	65.0 ± 21.4	0.478	61.3 ± 32.2	60.1 ± 14.0	0.909
EF (%)	64.6 ± 6.3	64.4 ± 5.8	0.906	64.9 ± 6.7	62.2 ± 9.1	0.194	61.2 ± 5.0	63.8 ± 5.8	0.261
ε_cc_ (%)	-14.3 ± 1.6	-13.7 ± 1.5	0.135	-13.4 ± 1.7	-12.1 ± 1.6	0.007	-13.2 ± 1.8	-11.9 ± 2.7	0.179

## Conclusions

In a large consecutive database of DMD patients, serial CMR studies demonstrate that current and standard treatment strategy at our institution has little effect on DMD-associated cardiac disease. The failure of current regimens supports the need for rigorous prospective clinical trials to identify effective treatment regimens.

